# Downregulation of urokinase plasminogen activator receptor expression inhibits Erk signalling with concomitant suppression of invasiveness due to loss of uPAR–*β*1 integrin complex in colon cancer cells

**DOI:** 10.1038/sj.bjc.6601098

**Published:** 2003-07-15

**Authors:** N Ahmed, K Oliva, Y Wang, M Quinn, G Rice

**Affiliations:** 1 Department of Obstetrics and Gynaecology, Gynaecological Cancer Research Centre, Royal Women's Hospital, University of Melbourne, Melbourne 3053, Australia; 2 Orthopaedic Research Institute, St George Hospital, University of New South Wales, Kogarah, Sydney, NSW 2217, Australia

**Keywords:** urokinase plasminogen activator receptor, *β*1 integrin, colon cancer, plasminogen activation and metastasis

## Abstract

Cancer invasion is regulated by cell surface proteinases and adhesion molecules. Interaction between specific cell surface molecules such as urokinase plasminogen activator receptor (uPAR) and integrins is crucial for tumour invasion and metastasis. In this study, we examined whether uPAR and *β*1 integrin form a functional complex to mediate signalling required for tumour invasion. We assessed the expression of uPAR/*β*1 integrin complex, Erk signalling pathway, adhesion, uPA and matrix metalloproteinase (MMP) expression, migration/invasion and matrix degradation in a colon cancer cell line in which uPAR expression was modified. Antisense inhibition of the cell surface expression of uPAR by 50% in human colon carcinoma HCT116 cells (A/S) suppressed Erk-MAP kinase activity by two-fold. Urokinase plasminogen activator receptor antisense treatment of HCT116 cells was associated with a 1.3-fold inhibition of adhesion, approximately four-fold suppression of HMW-uPA secretion and inhibition of pro-MMP-9 secretion. At a functional level, uPAR antisense resulted in a four-fold decline in migration/invasion and abatement of plasmin-mediated matrix degradation. In empty vector-transfected cells (mock), uPA strongly elevated basal Erk activation. In contrast, in A/S cells, uPA induction of Erk activation was not observed. Urokinase plasminogen activator receptor associated with *β*1 integrin in mock-transfected cells. Disruption of uPAR–*β*1 integrin complex in mock-transfected cells with a specific peptide (P25) inhibited uPA-mediated Erk-MAP kinase pathway and inhibited migration/invasion and plasmin-dependent matrix degradation through suppression of pro-MMP-9/MMP-2 expression. This novel paradigm of uPAR–integrin signalling may afford opportunities for alternative therapeutic strategies for the treatment of cancer.

Urokinase plasminogen activator receptor (uPAR) is the cellular receptor for urokinase, a serine proteinase that is constitutively or inducibly secreted by most uPAR-expressing cells (Chapman *et al*, 1999). Urokinase plasminogen activator receptor/CD87 is a glycoprotein (*M*_r_ 35 000–65 000) anchored to the plasma membrane by a glycosyl phosphatidylinositol (GPI) linkage (Blasi, 1999). Receptor-bound uPA converts plasminogen to plasmin that mediates pericellular proteolysis of extracellular matrix proteins. Urokinase plasminogen activator receptor is overexpressed by some cancer cells and its expression has been shown to correlate with poor prognosis of many cancers (Dano *et al*, 1999). In murine tumour models, expression or administration of uPAR antagonists has a marked inhibitory effect on the metastatic ability of cancer cells (Crowley *et al*, 1993) and on the growth of the primary tumour (Min *et al*, 1996). In a number of cancers, the expression of uPAR is required for invasive phenotype (Cohen *et al*, 1991). Overexpression of uPAR in human osteosarcoma cells facilitates matrix degradation (Kariko *et al*, 1993). Rats injected with breast cancer cells overexpressing uPAR, develop significantly large primary and metastatic tumours compared to control rats receiving breast cancer cells with lower numbers of uPAR (Xing and Rabbani, 1996). Down-regulating uPAR expression by antisense treatment inhibits glioma invasion through Matrigel (Mohan *et al*, 1999b) and invasion and metastasis by non-small-cell lung cancer cell line (Mohan *et al*, 1999a). Similarly, injection of adenovirus-uPAR construct into U87-MG tumours in nude mice caused regression of tumours (Ganesh *et al*, 1994). In colon cancer, a high uPAR expression portends a low 5-year survival rate (Ganesh *et al*, 1994). These findings clearly suggest that uPAR may be an important target in preventing cancer spread.

Urokinase plasminogen activator receptor has been reported to associate with many signalling molecules and to mediate signal transduction (Yebra *et al*, 1996; Aguirre Ghiso *et al*, 1999a; Wei *et al*, 1999). As uPAR has no transmembrane structure, the exact mechanism of uPAR-mediated cellular signalling remains unknown. The existence of one or more hypothetical ‘transmembrane adapter molecules’ that couples uPAR to other signalling molecules inside the cells has been proposed (Wei *et al*, 1999). It has been shown that *β*1, *β*2 and *β*3 integrin receptors interact with uPAR (Bohuslav *et al*, 1995; Todd and Petty, 1997; Xue *et al*, 1997; Degryse *et al*, 2001; Wei *et al*, 2001). The uPAR–integrin interaction is crucial as many integrin receptors activate intracellular signals coupled to pathways used by receptor and nonreceptor tyrosine kinases (Wary *et al*, 1996,^1998^). Integrin and receptor tyrosine kinases-mediated signals may complement each other to activate fully cell survival and proliferation pathways (Miyamoto *et al*, 1996; Yamada and Geiger, 1997). Recently, we have shown that the cytoplasmic domain of *α*v*β*6 integrin interacts with Erk and that interaction is essential to maintain tumour growth in mice (Ahmed *et al*, 2002a).

The uPAR–integrin complex can be specifically disrupted by a 17-amino–acid peptide sequence (P25), isolated from a phage peptide library (Wei *et al*, 1996). The P25 peptide decreased uPAR-mediated adhesion of embryonic kidney 293 cells to vitronectin, a substrate for uPAR when associated with integrins (Wei *et al*, 1996). Consistent with this observation, P25 peptide decreased adhesion of metastatic MDA-MB-231 breast cancer cell line to vitronectin, but increased its adhesion to fibronectin (van der Pluijm *et al*, 2001). Moreover, stable transfected MDA-MB-231 cells that overexpress P25 showed a significant reduction in tumour progression in bone, and was consistent with reduced tumour progression of MDA-MB-231 cells to bone by continuous administration of P25 peptides (van der Pluijm *et al*, 2001). These data suggest that adhesive and proteolytic events are tightly associated in cancer cells and functional uPAR–integrin complexes are involved in the processes.

In this study, we have investigated the mechanism by which a reduction in cell surface expression of uPAR impairs uPA/plasmin-mediated matrix degradation and invasion. We demonstrate that suppression of uPAR expression in a colon HCT116 cell line results in reduced activation of the Erk-MAP kinase pathway, leading to reduced HMW-uPA secretion, reduced adhesion, migration/invasion and plasminogen-dependent matrix degradation. Our result also demonstrates that downregulation of uPAR results in loss of uPAR/*β*1 integrin complex, resulting in abatement of uPA signalling through the uPAR/*β*1 integrin complex. Similar scenario is observed when uPAR/*β*1 integrin complex is disrupted by a specific P25 peptide. Even though the role of uPA/uPAR/integrin signalling in maintaining cell adhesion and proliferation has been described previously (Aguirre Ghiso *et al*, 1999b), our data for the first time establishes the role of this interaction in maintaining the invasive phenotype of colon cancer cells.

## MATERIALS AND METHODS

### Antibodies and reagents

Monoclonal antibody against uPAR (3936) was obtained from American Diagnostica (Greenwich, USA), antiphospho-Erk from New England Biolab (MA, USA) and pan-Erk from Santa Cruz (CA, USA). The monoclonal antibodies against integrin *α*1 (FB12), *α*2 (PIE6), *α*3 (PIB5), *α*4 (PIH4), *α*5 (PID6), *α*6 (CLB-701), *α*v (LM 142) and *β*1 (P5D2), were from Chemicon International (CA, USA). Phycoerythrin-conjugated goat anti-mouse IgG was obtained from Chemicon International (CA, USA) and peroxidase-conjugated goat anti-mouse antibody from Bio-Rad (CA, USA). Amiloride, 1,10-phenanthroline and Trasylol were purchased from Sigma (St Louis, USA). The Glu-plasminogen (glu-Plg) was from Calbiochem (Darmstadt, Germany) and Ukidan was obtained from Serono (Sydney, Australia).

### Peptide synthesis

Two synthetic peptides were used in the study: P25, AESTYHHLSLGYMYTLN-NH2 and a peptide with the exact composition of amino acids of P25 but in scrambled sequence order (Scp), NYHYLESSMTALYTLGH. Peptides were synthesised by Auspep Pty Limited (Parkville, Australia) and had a purity of greater than 95% as determined by reversed-phase HPLC.

### Preparation of uPAR-antisense constructs

The 5′ uPAR cDNA fragments were obtained from the 1.144 kb human uPAR cDNA. The fragment (*Xba*I/*Bam*HI, 498 bp) of the uPAR cDNA was subcloned into the expression vector pDR2 (Clontech) in an antisense orientation. The orientation of the antisense insert in the vector was confirmed by DNA sequencing. This was determined from the vector template using standard dye-labelled primer protocols from Applied Biosystems and a model 373A automated DNA sequencing system (Applied Biosystems, Inc., CA, USA). Analysis of DNA sequencing data and alignments of DNA sequences were performed using the MacVector program (Version 4.0.1, dated 1992, International Biotechnologies, Inc., New Haven, USA).

### Transfection and selection of the HCT116 clones

Cells (2 × 10^6^) were plated per 100 mm dish in Dulbecco's modified Eagle's medium (DMEM) with 10% FCS for 16 h and processed according to the protocol provided by Stratagene (CA, USA). The cells were transfected with 20 *μ*g of either antisense vector or empty vector using the calcium phosphate precipitation method. After incubation at room temperature for 20 min, the suspension was added dropwise to the above dishes and the cells cultured for further 48 h and then kept in DMEM with G418 and/or hygromycin. After 5 days, the antibiotic-resistant clones were isolated, subcultured and passaged. More than 400 antibiotic-resistant colonies were obtained and screened for either antisense-uPAR or full-length uPAR integration by Southern blot analysis. The clone with the most potent suppression of uPAR XT11'17 (A/S) was used for further studies in comparison to the parental HCT116 [wild-type (WT)] and an empty-vector-transfected clone (mock), XT0.

### Southern blot analysis

High molecular weight DNAs extracted from transfected or untransfected cells were electrophoresed, and further processed (Ausubel *et al*, 1987). The uPAR cDNA was labelled with (*α*-^32^P)-dCTP and hybridisation was carried out.

### Flow-cytometric analyses

Monolayer cultures of HCT116 cell lines (WT, mock and A/S) were washed twice with phosphate-buffered saline (PBS), harvested with trypsin-versene (CSL Biosciences, Australia) and 10^6^ cells were incubated with primary antibody for 30 min at 4°C and washed twice with phosphate buffered saline PBS. Cells were stained with secondary antibody conjugated with phycoerythrin for 20 min at 4°C, washed twice with PBS and then resuspended in 0.5 ml PBS prior to FACScan analysis (Becton Dickinson, NJ, USA). All data were analysed using Cell Quest software (Becton-Dickinson, NJ, USA). Results are expressed as mean fluorescence units for triplicate determinations.

### Cell proliferation and adhesion assays

In all, 1 × 10^4^ (proliferation assay) or 1 × 10^5^ (adhesion assay) mock and A/S HCT116 cells were plated on a specific protein-coated wells of a 96-well plate (Nunc, USA). For proliferation assay, after 24 h incubation at 37°C, MTT (5 mg ml^−1^) was added and cells were further incubated for 2 h. After removing the medium from the top, cells were solubilised in 1% SDS and absorbance was measured at 595 nm with a *V*_max_ plate reader (Bio-Rad, CA, USA).

For adhesion assays, cells were incubated in their respective protein-coated plates at 37°C for 90 min and then washed extensively with PBS to remove nonadhering cells. The adherent cells were fixed with 100% methanol for 5 min at room temperature, stained with 0.5% crystal violet for 15 min and then solubilised with 0.1% SDS. Absorbance at 595 nm was measured with *V*_max_ plate reader (Bio-Rad, CA, USA).

### Erk-MAP kinase assay

Monolayer cultures of colon cancer cell lines were washed twice with PBS and harvested with trypsin-versene (CSL Biosciences, Australia). Cells were counted by the Trypan blue exclusion method using a haemocytometer and disrupted using a sonicator (Ultrasonic Processor, USA). Three pulses of 30 s were used to sonicate cells with an interval of 30 s in between. Erk-MAP kinase assay was performed using an MAP kinase assay system (Amersham, UK). The ability of cell-derived sonicates to transfer phosphate from [*γ*^32^P] ATP to a synthetic peptide containing a p42/44 MAP kinase-specific phosphorylation site was quantitated. ^32^P-labelled peptides were spotted onto PEI-cellulose paper, nonpeptide bound radioactivity was removed by washing with 75 mM phosphoric acid and bound ^32^P-labelled peptides were measured by liquid scintillation counting. Protein estimation was performed on each cell sonicate used and enzyme activity calculated as described in the manufacturer's instructions. Cell lysates were also analysed by Western blotting. To test the effect of uPA on Erk activation, subconfluent cultures of cells were serum-starved overnight, washed and acid-stripped (0.05 M glycine-HCl in 0.1 M NaCl, pH 3) for 1 min to remove uPAR-bound uPA. The pH of the culture medium was neutralised quickly with two to three washes of serum-free DMEM medium. The cells were incubated in the presence of scuPA (20 nM) for 30 min. In some experiments, the cells were treated with P25 peptide (100 *μ*M) for 1 h before acid stripping. Controls were treated with the scrambled version of the peptide. In each case, the cell viability was checked by the Trypan blue exclusion method and found to be >95%.

### Preparation of conditioned medium

Conditioned medium was prepared as described previously (Ahmed *et al*, 2002c). Briefly, exponentially growing adherent cells were washed free of FBS and maintained in FBS-free medium. After 48 h, conditioned medium was concentrated 90–120-fold using Biomax Ultrafree Centrifugal Filter Unit (Millipore, Bedford, USA) with a 10-kDa pore diameter cutoff. In some experiments, cells were treated with P25 peptide (100 *μ*M) for 48 h. Control cells contained equal concentration of the scrambled peptide. The viability of cells was determined by Trypan blue exclusion method and found to be >95%.

### Western blotting

Conditioned medium and cell lysates containing equal amounts of protein were electrophoresed on 10% SDS–PAGE gels under nonreducing conditions and transferred to nitrocellulose membranes. Membranes were probed with primary antibody followed by peroxidase-labelled secondary antibody and visualised by the (ECL) (Amersham, UK) detection system according to the manufacturer's instructions.

### Zymography

Pro-MMP-2 and pro-MMP-9 expression in conditioned medium was analysed using 10% SDS-gelatin (1 mg ml^−1^ final concentration) zymography under nonreducing conditions as described previously (Ahmed *et al*, 2002c). Gelatinolytic activity attributed by MMP-2 and MMP-9 was confirmed by activation with APMA (2 mM) for 4 h prior to zymography. Activation of pro-MMP-2 and pro-MMP-9 could be abolished by incubating zymograms with 1 : 10 phenanthroline (2 mM) or EDTA (data not shown).

### Preparation and degradation of collagen type IV basement membrane

HCT116 cell lines (WT, mock and A/S) were plated directly onto the isotopically labelled collagen IV matrix at a density of 3 × 10^5^ cells well^−1^ in 0.3 ml serum-free medium as described previously (Ahmed *et al*, 2002c). After 24 h, the conditioned medium was removed, centrifuged in a Beckman microfuge for 5 min and counted in a *β*-scintillation counter to monitor release of soluble [^3^H]-collagen fragments from the insoluble matrix. Matrix degradation of the cells was measured by the release of [^3^H]-collagen in the medium. In studies involving inhibition of plasminogen activation, uPA and MMP activity, cells were treated with inhibitors, antibodies and peptides (P25 and Scp) 30 min prior to plating onto the [^3^H]-collagen-labelled matrix. Cell viability was checked by Trypan blue exclusion method and found to be >95%.

### Migration and invasion assay

The migratory and invasive response of HCT116 cell lines was determined using Matrigel-coated invasion chambers (Becton and Dickinson, USA). Approximately 2 × 10^5^ cells were incubated in Matrigel-coated inserts in serum free medium. Fetal bovine serum (10%) was used as chemoattractant at 37°C for 36 h. Matrigel inserts were removed and 0.5 *μ*Ci of [^3^H]-thymidine was added to the cells in the wells for 12 h. Cells were washed with PBS, dissolved in 1% Triton and measured by liquid scintillation counting. The migration and invasion of the cells was measured by [^3^H]-thymidine uptake of the cells migrating from the inserts into the wells. In some experiments, cells were treated with peptides (P25 and Scp) in Matrigel inserts. Cell viability was checked by Trypan blue exclusion method and found to be >95%.

### Immunoprecipitation and coimmunoprecipitation analyses

Monolayer cultures of HCT116 cell lines were washed twice with PBS and harvested with trypsin-versene. To detect cell surface expression of uPAR or *β*1 integrin, cells were biotinylated at 4°C for 40 min and then lysed in lysis buffer (100 mM Tris-HCl, pH 7.5, 150 mM NaCl, 1 mM CaCl_2_, 1% Triton X-100, 0.1% SDS, 0.1% NP-40, 1 mM vanadate, 1 *μ*g/ml^−1^ pepstatin, 1 mM PMSF, 5 *μ*g ml^−1^ Trasylol and 1 *μ*g ml^−1^ of leupeptin). The insoluble fraction of the cell lysate was further solubilised in 1% Triton X-100. Total cells lysates were immunoprecipitated with mAbs against uPAR (3936, ADI) or *β*1 (PD52, Chemicon), or isotype-matched control. Samples were resolved in 10% SDS–PAGE gel under nonreducing conditions and were transferred to nitrocellulose membranes. In case of biotinylation, membranes were probed with streptavidin–HRP and then evaluated by ECL. In other cases, membranes were probed with anti-uPAR or *β*1 integrin antibody followed by peroxidase-labelled secondary antibody. ECL was used to detect the protein bands. In some experiments, cell lysates were immunodepleted of uPAR or *β*1 integrin after five rounds of sequential uPAR or *β*1 immunoprecipitation.

### Statistical methods

Student's *t*-test was used for statistical analyses. Statistical significance was indicated by *P*<0.05. Data are presented as means±S.E.M.

## RESULTS

### Characterisation of untransfected and transfected HCT116 cells

**Figure 1:**
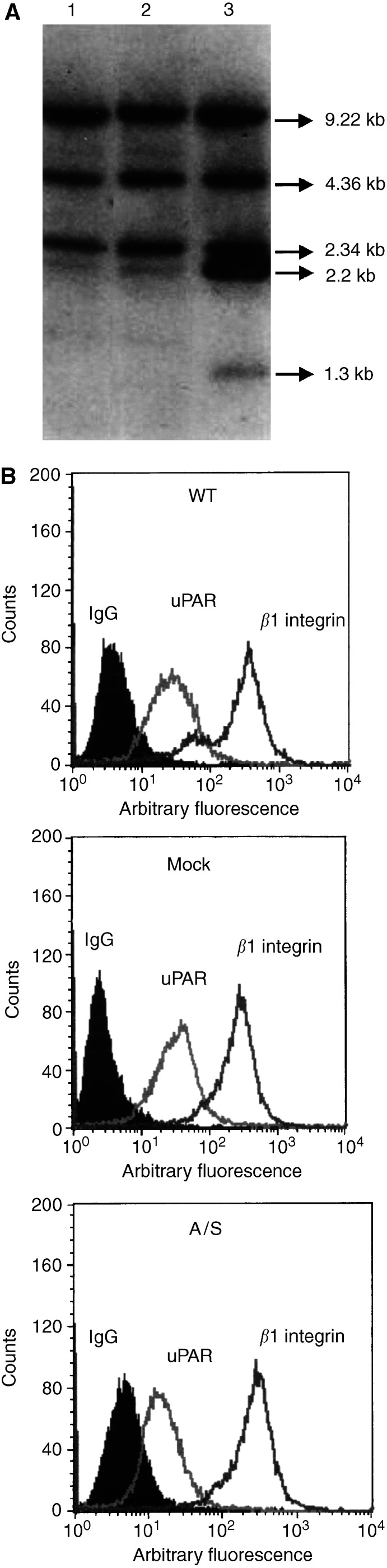
Characterisation of HCT116 cells. (**A**) Southern blot analyses of genomic DNAs isolated from transfected and untransfected HCT116 cells. Lane 1, WT HCT116 cells; lane 2, mock-transfected HCT116 cells; and lane 3 is 5′A/S HCT116 cells. (**B**) Flow-cytometric analyses of uPAR and *β*1 integrin in HCT116 cell lines. The median intensity of fluorescence (MIF, arbitary units, log scale) was measured. Results are representative of three independent experiments.

To confirm the integration of antisensense uPAR cDNA into the genome of the cells, Southern blot analyses were performed. As shown in Figure 1

A parental (WT) and empty-vector-transfected cells (mock) had three main bands of 9.22, 4.36 and 2.34 kb (lanes 1 and 2). The antisense-transfected cells (A/S) revealed additional bands of 2.2 and 1.3 kb (lane 3). The extra bands hybridised to the uPAR cDNA provided evidence of integration of one or more copies of exogenous uPAR cDNA into the genome of the cells.

The patterns of cell surface expression of uPAR and *β*1 integrin subunit on HCT116 clones were assessed by flow cytometry using anti-uPAR and anti-*β*1 integrin antibody. The expression of cell surface uPAR in WT and mock-transfected cells was two-fold higher than in A/S clone, but there was no change in the expression of *β*1 integrin (Figure 1B). There was no difference in the proliferative response of mock- and A/S-transfected cells (Table 1

**Table 1:** Effect of uPAR suppression on the proliferative and adhesive response of HCT116 cell lines

**Cell type**	**MTT response (absorbance at 595 nm)**	**Adhesive response (absorbance at 595 nm)**
HCT116 (mock)	1.54±0.16	0.74±0.04
HCT116 (A/S)	1.34±0.22	0.58±0.01*

Results are shown as mean±S.E.M of three different experiments performed in triplicat.

* Significantly different, *P*<0.002, compared to mock-transfected cells.

). The adhesive response of HCT116 clones was assessed on poly-L-lysine, fibronectin and vitronectin. The suppression of uPAR expression decreased the adhesion of A/S cells on poly-L-lysine by 1.3-fold (Table 1). Similar effects were observed on fibronectin or vitronectin (data not shown).

### Suppression of uPAR decreases activation of Erk MAP kinase in HCT116 cells

As constitutive activation of Erk-MAP kinase has been implicated with upregulation of uPAR in several human tumour types (Hoshino and Kohno, 2000), we investigated whether p42/44 Erk-MAP kinase activity plays a role in the regulation of uPAR expression in HCT116 cells. *In vitro* kinase assay, specific for p42/44 MAP kinase activity, showed approximately two-fold higher MAP kinase activity in WT and mock-transfected HCT116 cell lines compared to A/S cell line (Table 2

**Table 2:** Effect of uPAR suppression on the MAP kinase activity of HCT116 cell lines

**Cell type**	**MAP kinase activity (pmol min^−1^ mg^−1^ of protein)**
HCT116 (WT)	337±41
HCT116 (mock)	290±33
HCT116 (A/S)	140±22*

Results are presented as mean±S.E.M of three different experiments performed in triplicate.

**Figure 2:**
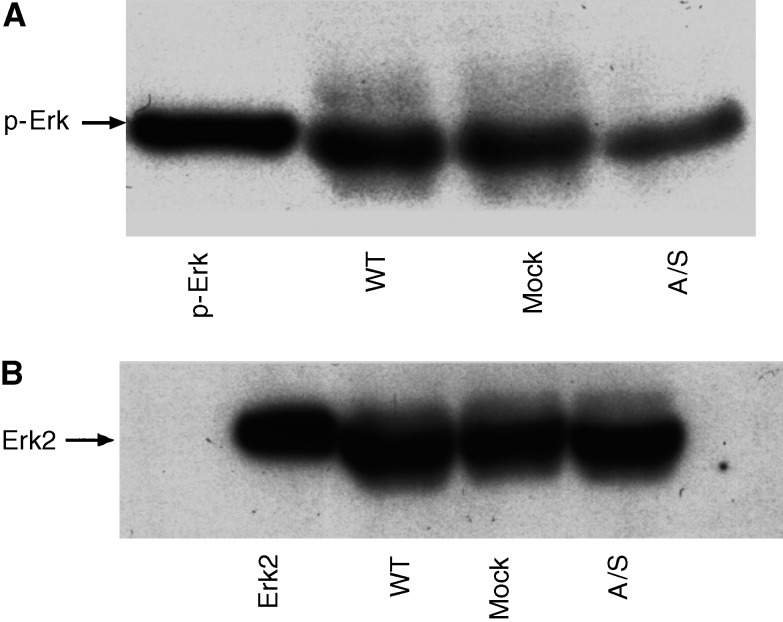
(**A**) Erk-MAP kinase activity in HCT116 cell lines. Cell lysates used for *in vitro* kinase assay were analysed by equal protein loading on 10% SDS–PAGE (under nonreducing conditions) followed by Western blotting using antibodies against phosphorylated Erk-MAP kinase (E10 antibody, New England Biolabs) and (**B**) total Erk (Santa Cruz, CA, USA).

* Significantly different, *P*<0.001, compared to WT and mock-transfected cell lines.

). Results from *in vitro* kinase assay were consistent with Western blotting using antibodies against phospho-Erk that showed inhibition of phospho-Erk expression in A/S cell line (Figure 2A

), but there was no change in total immunoreactive Erk content as determined by pan-Erk antibody (Figure 2B).

### Suppression of uPAR correlates with decreased secretion of HMW-uPA and abrogates the secretion of pro-MMP-9 into the conditioned medium

**Figure 3:**
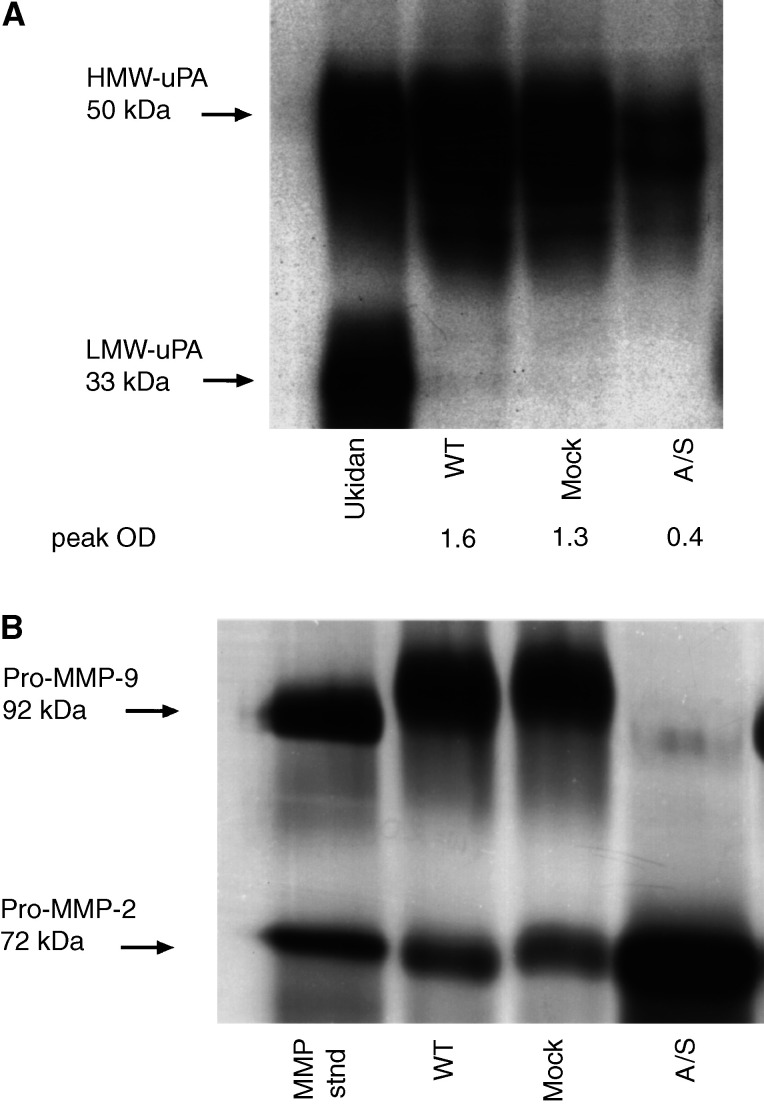
(**A**) Expression of HMW-uPA in conditioned medium of WT, mock- and A/S-transfected HCT116 cell lines. Conditioned medium was analysed by equal protein loading on 10% SDS–PAGE (under nonreducing conditions) followed by Western blotting using monoclonal anti-uPA antibody. Ukidan was used as standard reference uPA (Serono, Australia). The expression of HMW-uPA was quantified by densitometry and is expressed as peak optical density (peak OD). Results are representative of three experiments. (**B**) Gelatin zymography showing the amounts of pro-MMP-2 and pro-MMP-9 secreted in conditioned medium (concentrated 90–100-fold) from HCT116 cell lines. The positions of purified pro-MMP-2 and pro-MMP-9 are shown on the left. Results are representative of three independent experiments.

In order to evaluate a potential role of uPAR in the regulation of proteolytic system, we examined the expression of HMW-uPA, pro-MMP-2 and pro-MMP-9 in the conditioned medium from HCT116 cell lines. Western blotting of conditioned medium with equal protein loading showed approximately four-fold higher expression of HMW-uPA in the conditioned medium of WT and mock-transfected cells compared to that in A/S cells (Figure 3A

). Gelatin zymography on conditioned medium showed high expression of pro-MMP-9 in WT and mock-transfected HCT116 cells. Although A/S cell line showed high expression of pro-MMP-2, the expression of pro-MMP-9 was absent (Figure 3B). Collectively, these data demonstrated that the expression of uPAR strongly correlates with elevated expression of the cell surface proteinases.

### Suppression of uPAR correlates with decreased migration/invasion and plasminogen-dependent [^3^H]-collagen IV degradation

The possible correlation of uPAR expression with invasion and extracellular matrix proteolysis was determined in WT, mock and A/S HCT116 cell lines. For migration/invasion assay, invasion chambers coated with Matrigel (Becton and Dickinson, MA, USA) were used, while for matrix degradation [^3^H]-collagen IV substrate was chosen as substrate, as this molecule is the predominant collagen type in the basement membrane and is the substrate for both type IV collagenases (MMP-2 and MMP-9). WT and mock-transfected cells showed four-fold greater migratory/invasive capacity by Matrigel invasion assay compared to A/S cell lines (Table 3

**Table 3:** Effect of uPAR suppression on the migration/invasion capacity of HCT116 cell lines

**Cell type**	**[^3^H]-thymidine uptake**
HCT116 (WT)	3300±98
HCT116 (mock)	3600±88
HCT116 (A/S)	888±48*

The migration and invasion of the cells were measured by [^3^H]-thymidine uptake of the cells migrating from the inserts into the wells. Results are shown as mean±S.E.M of three different experiments performed in triplicate.

* Significantly different, *P*<0.001, compared to WT and mock-transfected cells.

). Even though the basal matrix degradation capacity in the absence of Plg was approximately the same, the mock-transfected clone had a two-fold enhanced capacity to degrade [^3^H]-labelled collagen IV when plasminogen was present (Table 4A

**Table 4A:** (a): Effect of uPAR suppression on [^3^H]-collagen IV degradation in HCT116 cell lines

Cell type	Matrix degradation (CPM/3 × 10^5^ cells)	
	−Plg	+Plg
HCT116 (mock)	275±32	600±56*
HCT116 (A/S)	264±30	308±22

Matrix degradation of the cells was measured by the release of [^3^H]-collagen IV in the medium. Results are shown as mean±S.E.M of three different experiments performed in triplicate.

* Significantly different, *P*<0.001, compared to control cells in the absence of Plg.

). In contrast, the addition of Plg to A/S clone produced no increase in the degradation of basement membrane. The two-fold enhanced Plg-dependent collagen IV degradation seen in mock HCT116 cell line was abolished by the addition of either uPA inhibitor amiloride, the zinc chelator 1,10 phenanthroline, plasmin inhibitor Trasylol and anti-uPAR antibody (Table 4B

**Table 4B:** (b): Effect of uPA, MMPs and plasmin inhibitors on the matrix degradation of mock-transfected HCT116 cell line

**Cell treatment**	**Matrix degradation (CPM/3 × 10^5^ cells)**
−Plg	275±32
+Plg	600±56
+Plg+DMSO	586±37
+Plg+amiloride (2 mM)	263±42*
+Plg+phenanthroline (2 mM)	260±33*
+Plg+Trasylol (10 *μ*g ml^−1^)	270±30*
+Plg+IgG2a (10 *μ*g ml^−1^)	586±60
+Plg+anti-uPAR (10 *μ*g ml^−1^)	426±40*

Matrix degradation of the cells was measured by the release of [^3^H]-collagen IV in the medium. Results are shown as mean±S.E.M of three different experiments performed in triplicate.

* Significantly different, *P*<0.001, compared to control cells in the presence of Plg.

). These data strongly suggest that in colon cancer uPA-mediated plasmin-dependent serine and metalloproteolytic activity requisite for matrix degradation coinside with high uPAR expression and can be inhibited by blocking uPA/uPAR and plasmin mediated pathways.

### Suppression of uPAR impairs the uPA-induced Erk activation in HCT116 cells

**Figure 4:**
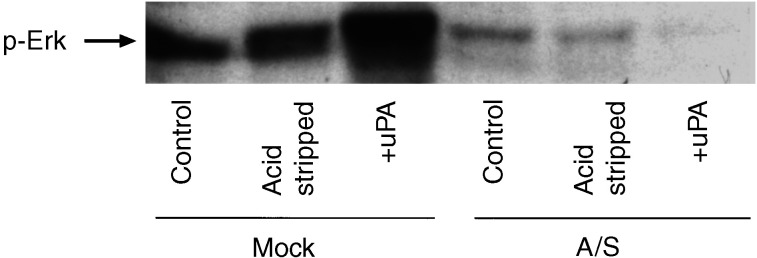
Effect of uPA on the activation of Erk. Subconfluent cultures of mock and A/S HCT116 cells were serum starved for 24 h, acid stripped for 1 min and incubated with uPA (20 nM) for 30 min. The level of phospho Erk1/2 was determined by Western blot using equal protein loading. Results are representative of one experiment. The experiment was repeated three times.

In cells, 80–90% of uPAR is occupied by endogenously produced uPA (Ossowski, 1992), and uPA binds to uPAR to initiate signal transduction (Nguyen *et al*, 1998). We tested whether or not the addition of exogenous uPA had any affect on the signal to Erk activation. Mock- and A/S-transfected HCT116 cells stripped of endogenous uPA were incubated for 30 min with uPA (20 nM). uPA induced a strong increase in phospho-Erk levels, while no such stimulation was detected in A/S cells (Figure 4

). These results suggest that low level of Erk activation in A/S HCT116 cells is the result of a reduced uPA/uPAR signalling capacity to endogenous uPA.

### *α* subunit integrin profile of mock- and A/S-transfected HCT116 cells

As *β*1 integrin subunit binds with different *α* subunits, we wanted to determine if suppression of uPAR expression had any effect on the cell surface expression of α integrin subunits in HCT116 cell lines. The pattern of cell surface expression of α integrin subunits was assessed using a panel of integrin-specific antibodies (Table 5

**Table 5:** Cell surface expression at *α* integrin subunits in mock and A/S HCT116 cell lines

**Cell type**	***α*2 integrin MIF**	***α*3 integrin MIF**	***α*6 integrin MIF**	***α*v integrin MIF**
HCT116 (mock)	164	225	23	48
HCT116 (A/S)	184	224	20	60

MIF=mean intensity of fluorescence.

). Both cell lines expressed *α*v, *α*2, *α*3 and *α*6 integrin subunits. Suppression of uPAR had no effect on integrin subunit expression.

### Suppression of uPAR results in loss of uPAR/*β*1 integrin complex

**Figure 5:**
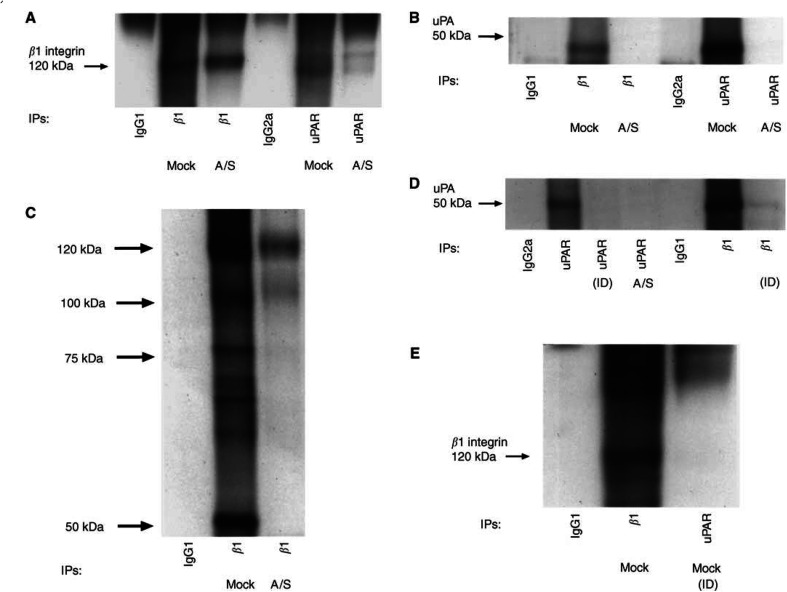
Coimmunoprecipitation of *β*1 integrin and uPAR. Mock- and A/S-transfected HCT116 cells were lysed in Triton X-100 buffer (see Materials and Methods). Cell protein (500 *μ*g) was mixed with anti-*β*1 antibody (PD52) or anti-uPAR antibody (3936) or isotype-matched mouse IgG and the resulting immunoprecipitates were analysed by Western blotting with (**A**) anti-*β*1-integrin antibody (PD52) or (**B**) anti-uPAR antibody (3936). (**C**) Cells were surface biotinylated and cell extracts were subjected to immunoprecipitation with anti-*β*1 antibody or with isotype-matched IgG antibodies and analysed by streptavidin–HRP binding to biotinylated proteins after SDS–PAGE and transfer to nitrocellulose membranes. (**D** and **E**) Mock-transfected cell lysates were immunodepleted (ID)of *β*1 after five rounds of sequential *β*1 and uPAR immunoprecipitation. Cell lysates were resolved by 10% SDS–PAGE followed by blotting with (**D**) anti-uPAR and (**E**) anti-*β*1 integrin antibody. The experiments were repeated at least three times.

uPAR and *β* integrin interaction result in integrin activation in leucocytes during *in vivo* transendothelial migration (May *et al*, 1998) and in modulating integrin function as adhesion receptors in 293 kidney and ovarian cancer cells (Wei *et al*, 1996; Hapke *et al*, 2001). Therefore, we tested whether change in adhesion and invasive function of A/S HCT116 cells due to 50% reduction in uPAR expression had any effect on uPAR/*β*1 interaction. We first examined whether uPAR and *β*1 integrin were physically associated by testing the ability of anti-*β*1 antibody to coimmunoprecipitate (co-IP) uPAR and of uPAR antibody to co-IP *β*1 integrin from cell lysates. In mock-transfected HCT116 cells, immunoprecipitates (IPs) of *β*1 integrin and uPAR revealed a complex that was absent in A/S cells reflecting, most likely, the low levels of uPAR in these cells (Figures 5A,B

). Even though, both mock-and A/S-transfected cells contained similar amounts of *β*1 integrin (Figure 1B) interacting band with uPAR was drastically diminished in A/S cell line. In another approach, cells were biotinylated before immunoprecipitation with *β*1 integrin antibody. In mock-transfected cells, anti-*β*1 antibody coimmunoprecipitated bands at 50 and 120 kDa corresponding to uPAR and *β*1 integrin respectively (Figure 5C) (two unidentified bands at 90–100 and 75–80 kDa were also present). On the other hand, *β*1 integrin IP in A/S cells showed *β*1 integrin corresponding band, but the band corresponding to uPAR was reduced dramatically (Figure 5C). The absence of other unidentified bands at 75–80 and 90–100 kDa in *β*1 integrin IP of A/S-transfected cells indicates that downregulation of uPAR results in loss of complex formed by *β*1 integrin with other proteins. The identity of uPAR and *β*1 integrin interaction was verified further in mock-transfected HCT116 cells by immunodepleting the cell lysates of *β*1 integrin (Figures 5D and 5E) or uPAR (Figure 5E) by five rounds of immunodepletion steps. This resulted in loss of uPAR or *β*1 integrin interacting band indicating that uPAR directly interacts with *β*1 integrin in HCT116 cells.

### Functional role of uPAR/*β*1 integrin complex in mock-transfected HCT116 cells

**Figure 6:**
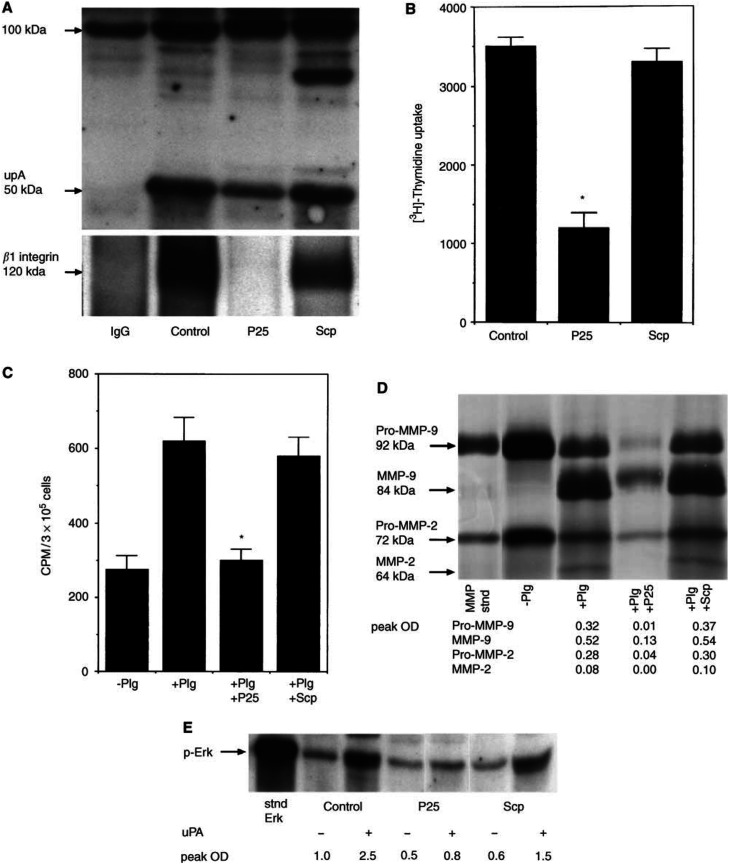
uPAR/*β*1 integrin complex and the affect of P25 peptide. (**A**) P25 peptide but not scrambled peptide (Scp) disrupts uPAR/*β*1 integrin complex in mock-transfected HCT116 cells. Cells were treated for 16 h with P25 peptide (100 *μ*M) and Scp (100 μM). Immunoprecipitates of uPAR were prepared and coimmunoprecipitated *β*1 integrin band was analysed. (**B**) Migration/invasion of HCT116 mock cells in the presence of P25 peptide and its scrambled analogue (100 *μ*M each). (**C**) Plasminogen-dependent matrix degradation of HCT116 mock cells in the presence of P25 peptide and its scrambled analogue (100 *μ*M each). Results for both Figures 6B and C are shown as mean + S.E.M. of three different experiments performed in triplicate (^*^P<0.001, compared to control cells in the presence of Plg). (**D**) Effects of P25 peptide and its scrambled analogue on the secretion and activation of pro-MMP-2/MMP-9. Conditioned medium in the presence and absence of Plg, P25 peptide and Scp (100 *μ*M each) was prepared as described in the Materials and Methods section. The samples were analysed by equal protein loading by gelatin-zymography for the activation of pro-MMP-2/MMP-9. Quantification of MMP secretion in the tumour-conditioned medium was performed by densitometry and the results are expressed as peak OD. Results are representative of one experiment. The experiment was repeated three times. (**E**) Effect of P25 peptide and its scrambled analogue (100 *μ*M each) on uPA-induced activation of Erk in mock-transfected HCT116 cells. Subconfluent cultures of mock-transfected HCT116 cells were serum starved for 24 h, acid stripped for 1 min and incubated with uPA (20 nM) for 30 min. The level of phospho Erk1/2 was determined by Western blot using equal protein loading. Quantification of phospho Erk1/2 expression was performed by densitometry and expressed as peak OD. Results are representative of one experiment. The experiment was repeated three times.

The functional role of uPAR/*β*1 integrin complex in terms of migration/invasion and matrix degradation was studied in HCT116 cells using a 17-amino-acid peptide sequence (P25) at 100 *μ*M concentration, a concentration that has been shown to disrupt specifically uPAR/*β*1 integrin complexes without affecting ligand binding of both receptors (Wei *et al*, 1996). In mock-transfected HCT116 cells, P25 peptide (100 *μ*M) disrupted uPAR/*β*1 integrin complex as shown by the loss of *β*1 interacting band in uPAR IP (Figure 6A

). P25 peptide (100 *μ*M) also inhibited migration/invasion (Figure 6B) and plasmin-mediated collagen IV degradation (Figure 6C) through suppression of pro-MMP-9 (three-fold) and pro-MMP-2 (seven-fold) secretion and lack of activation of plasmin-mediated pro-MMP/2 (Figure 6D).

We determined if uPA-mediated activation of Erk-MAP kinase signalling through uPAR/*β*1 integrin complex is required for pro-MMP-2/MMP-9 secretion and subsequent invasion and plasmin-mediated matrix degradation. Mock-transfected cells were acid stripped of endogenous uPA and then stimulated with uPA (20 nM) for 30 min in the presence of P25 peptide and its scrambled analogue. In control and Scp-treated cells, uPA induced 2.5-fold enhancement of Erk activation compared to only 1.6-fold in the presence of P25 peptide. Hence, P25 peptide, but not its scrambled analogue was able to inhibit uPA-induced activation of Erk (Figure 6E) suggesting that functional uPAR/*β*1 complex is required for optimum endogenous uPA-mediated Erk activation essential for plasmin-mediated matrix degradation.

## DISCUSSION

Previous reports have shown that suppression of cell surface uPAR expression induces a dormancy in carcinoma cells characterised by cancer cell survival but unaccompanied by increase in tumour mass (Yu *et al*, 1997). Recently, we have demonstrated inhibition of metastatic capability of colon cancer HCT116 cell line *in vivo* by inhibition of uPAR expression by an antisense method (Wang *et al*, 2001). This outcome is possibly due to reduced signalling by uPA through uPAR expressed below the threshold limit required to initiate an outside-in signalling cascade capable of maintaining the cancer cells in a proliferative and invasive state (Aguirre Ghiso *et al*, 1999b). Results reported here further develop this paradigm by elucidating the specificity of uPAR and *β*1 integrin interaction in terms of uPA/plasmin-mediated activation and subsequently matrix degradation. Our results demonstrate that downregulation of uPAR expression by an antisense approach in a colon cancer cell line was associated with a two-fold inhibition of Erk-MAP kinase pathway, inhibition of uPA secretion, complete inhibition of pro-MMP-9 secretion, 1.3-fold inhibition in adhesion, four-fold inhibition in migratory/invasive capacity and complete abrogation of plasmin-dependent matrix degradation. uPAR-suppressed cells have reduced uPA/uPAR signalling capacity and they lack the capacity to elicit Erk phosphorylation in response to low concentration of uPA. In mock-transfected uPAR-rich cells, uPAR interacts with *β*1 integrin and that interaction is drastically reduced in uPAR-suppressed A/S cells. We also demonstrate that uPAR/*β*1 integrin complex initiate signalling pathways promoting matrix degradation, as disruption of this complex results in suppression of pro-MMP-9/MMP-2 secretion and lack of activation of plasmin-mediated pro-MMP-2, resulting in total abrogation of matrix degradation. To our knowledge, this is the first study to report links between uPAR/*β*1 integrin complex, Erk signalling pathway and the expression and activation of metalloproteinases leading to matrix degradation.

There is abundant evidence in the literature indicating that uPA/uPAR interaction activates the Erk pathway (Ossowski and Aguirre-Ghiso, 2000). The cell type where these pathways are activated may have different biological outcomes such as migration and motility, growth and differentiation, etc. (Yebra *et al*, 1996; Stepanova *et al*, 1997; Dumler *et al*, 1999; Ferrero *et al*, 2000). As the binding of uPA to uPAR is critical for the activation of Erk, we can hypothesise that high level of endogenous uPA expressed and secreted by WT and mock-transfected HCT116 cells can bind to excess uPAR on the cell surface, and thereby regulate the steady-state level of active Erk. The fact that a low concentration of uPA induces a strong increase in active Erk levels in acid-stripped uPAR-rich mock-transfected cells while no such effect can be seen in uPAR-reduced A/S cells support this assumption and suggest that down regulation of uPAR-expression in cancer cells reduces the ability of the cells to initiate uPA-mediated Erk signalling. Our observations are consistent with those of Aguirre Ghiso *et al*. (1999b) who showed lack of responsiveness of human carcinoma Hep3 cells to uPA when cell surface expression of uPAR was reduced by 70%. Similarly, Webb *et al* (2000) showed that neutralisation of receptors in LDL receptor family (LRF) leads to inhibition of catabolism of uPA and uPAR, resulting in increased binding of endogenously produced uPA to uPAR and the enhancement of basal level of activated Erk. Moreover, recently it has been shown that endogenously produced uPA is a major determinant of the basal level of activated Erk in an aggressive breast cancer cell line (Ma *et al*, 2001). Hence, the ability of uPA/uPAR system to sustain elevated basal level of activated Erk may represent a mechanism whereby uPA/uPAR system may affect cancer progression and distant metastasis *in vivo.*

Our study also demonstrates that downregulation of uPAR abrogates plasminogen-dependent matrix degradation in low uPAR-expressing A/S HCT116 cells. Inhibition of Erk-MAP kinase pathway has been shown to affect the expression of serine proteinase uPA (De Kier Joffe *et al*, 2000) and metalloproteinase MMP-9 (Cho *et al*, 2000). We have recently shown that activation of Erk is critical for plasminogen-dependent matrix degradation mediated by pro-uPA and pro-MMP-9 (Ahmed *et al*, 2002b). Since, activation of MMPs by plasminogen occurs through uPA-dependent plasmin generation (Ramos-DeSimone *et al*, 1999), it can be suggested that enhanced expression of uPAR is critical for sustaining basal level of activated Erk requisite for the secretion of pro-MMPs and plasminogen-mediated activation of the proteinases in colon cancer cells.

As uPAR lacks the transmembrane signalling domain, we explored the involvement of *β*1 integrin as a possible ‘adapter molecule’ capable of initiating signalling of uPA through uPAR. We show that uPAR physically interacts with *β*1 integrin in uPAR-rich mock-transfected HCT116 cells and that interaction is significantly diminished in low uPAR-expressing A/S cells. The expression of *β*1 integrin and its associated *α*v, *α*2, *α*3 and *α*6 subunits remain unchanged with the downregulation of uPAR expression in A/S-transfected cells. Hence, one can postulate that it is not the integrin expression itself but its association with uPAR and other interacting molecules (caveolin, src kinases, nucleolin, casein kinase 2, etc.) that may affect phenotypic differences between the cell lines. Consistent with that, the activation of Erk-MAP kinase cascade has been shown to be generated by the combined effect of uPA/uPAR/*β*1 integrin complex if uPAR expression exceeds a certain threshold limit (Aguirre Ghiso *et al*, 1999b). If the interaction is diminished as in the case of downregulation of uPAR by antisense treatment or interrupted by inhibitors, the signalling complex is turned off. This is consistent with the effect of P25 peptide on Erk activation, pro-MMP-2/MMP-9 secretion and inhibition of matrix degradation. Hence, one can suggest that lateral interaction between uPAR and *β*1 integrin in uPAR-rich cells may render Erk pathway constitutively active to maintain the expression of proteases required for the invasive phenotype of cancer cells. Hence, we can hypothesise that uPAR-rich colon cancer cells endogenously produces its ligand uPA to sustain elevated level of Erk-MAP kinase pathway through uPAR/*β*1 integrin complex. Considering that elevated level of uPAR is associated with negative prognosis of colon cancer (Ganesh *et al*, 1994) and is observed in invasive colon tumours (Ganesh *et al*, 1994; Wang, 2001), the impact of uPAR/*β*1 integrin complexes in colon cancer invasion and metastasis needs to be evaluated. To the best of our knowledge, ours is the first study to report the mechanism(s) of invasiveness of cancer cells through uPA/uPAR/integrin binding.
